# CTCs Expression Profiling for Advanced Breast Cancer Monitoring

**DOI:** 10.3390/cancers11121941

**Published:** 2019-12-04

**Authors:** Thais Pereira-Veiga, Mónica Martínez-Fernández, Carmen Abuin, Roberto Piñeiro, Victor Cebey, Juan Cueva, Patricia Palacios, Cristina Blanco, Laura Muinelo-Romay, Alicia Abalo, Clotilde Costa, Rafael López-López

**Affiliations:** 1 Roche-Chus Joint Unit, Translational Medical Oncology Group, Oncomet, Health Research Institute of Santiago de Compostela, Travesía da Choupana s/n, 15706 Santiago de Compostela, Spain; thaispv85@gmail.com (T.P.-V.); Carmen.Abuin.Redondo@sergas.es (C.A.); roberto.pineiro.cid@sergas.es (R.P.);; 2 Genomes and Disease Lab, CIMUS-Molecular Medicine and Chronic Diseases Research Centre, Universidade de Santiago de Compostela, Avda Barcelona s/n, 15706 Santiago de Compostela, Spain; monicamartinezfernandez@gmail.com; 3 Department of Oncology, Complexo Hospitalario Universitario de Santiago de Compostela (SERGAS), 15706 Santiago de Compostela, Spain; victor.cebey.lopez@sergas.es (V.C.); patricia.palacios.ozores@sergas.es (P.P.); cristina.blanco.freire@sergas.es (C.B.); 4 CIBERONC, Centro de Investigación Biomédica en Red Cáncer, 28029 Madrid, Spain; 5 Liquid Biopsy Analysis Unit, Translational Medical Oncology Group, Health Research Institute of Santiago, de Santiago de Compostela (SERGAS), Trav. Choupana s/n, 15706 Santiago de Compostela, Spain; alicia.abalo.pineiro@sergas.es

**Keywords:** CTCs, breast cancer, metastasis, gene expression, molecular/gene signature, predictive biomarkers

## Abstract

The study of circulating tumor cells (CTCs) has a huge clinical interest in advance and metastatic breast cancer patients. However, many approaches are biased by the use of epithelial markers, which underestimate non-epithelial CTCs phenotypes. CTCs enumeration provides valuable prognostic information; however, molecular characterization could be the best option to monitor patients throughout the disease since it may provide more relevant clinical information to the physicians. In this work, we aimed at enumerating and performing a molecular characterization of CTCs from a cohort of 20 patients with metastatic breast cancer (MBC), monitoring the disease at different time points of the therapy, and at progression when it occurred. To this end, we used a CTC negative enrichment protocol that allowed us to recover a higher variety of CTCs phenotypes. With this strategy, we were able to obtain gene expression data from CTCs from all the patients. In addition, we found that high expression levels of *PALB2* and *MYC* were associated with a worse outcome. Interestingly, we identified that CTCs with an *EpCAM*^high^*VIM*^low^*ALDH1A1*^high^ signature showed both shorter overall survival (OS) and progression-free survival (PFS), suggesting that CTCs with epithelial-stem features had the most aggressive phenotype.

## 1. Introduction

Breast cancer (BC) is the leading cancer-related cause of death in women [1], being the metastatic disease responsible for most of these deaths [2]. It has been estimated that ∼6% of patients have metastatic disease at the time of diagnosis, and ∼20–50% of patients first diagnosed with primary breast cancer (BC) will develop metastatic disease [3]. The characterization of BC molecular subtypes has improved the disease management in last years; thus, this heterogeneous disease has been classified in three basic therapeutic groups according to the expression of the progesterone (PR) and/or estrogen (ER) receptors and the human epidermal growth factor receptor 2 (HER2). These molecular subtypes include the luminal A or B subtype (PR and/or ER-positive), which represents around two thirds of the total cases and is treated with endocrine therapy [4]; HER2 overexpressed subtype, whose treatment should include anti-HER2 therapy; and triple-negative BC (TNBC), which leads to a short disease-specific survival and poor prognosis, being the recommended systemic chemotherapy [5,6,7]. This classification is normally performed based on the molecular characterization of the primary tumor. However, once the metastatic setting is present, the molecular information from the primary tumor might not represent the current status of the disease.

Circulating tumor cells (CTCs) are directly involved in the metastatic process, and their presence in the blood is associated with poor prognosis [8]. In addition, CTCs can provide valuable information for the clinical management of cancer patients in terms of cancer characterization and monitoring, since peripheral blood sampling is safe and can be repeated when needed [9]. However, CTCs detection is hampered by their molecular heterogeneity and low ratio in the peripheral blood. Furthermore, the existence of tumor heterogeneity and the phenotypic changes promoted during the epithelial-mesenchymal transition (EMT) process hinder the selection of appropriate CTCs markers. The CellSearch^®^, which is the only FDA-approved method for CTC quantification, selects CTCs based on the Epithelial Cell Adhesion Molecule (EpCAM) expression, constituting an obstacle to quantify these cells in tumors with a more mesenchymal or stem phenotype [10]. Furthermore, although CTCs enumeration has prognostic value, it is not enough for the identification of therapeutic targets or resistance mechanisms [11]. Thus, understanding the biology of these cells through their molecular characterization can help the stratification of patients.

The aim of this study was to approach the characterization of CTCs throughout the advance BC disease, without any bias introduced by marker selection. This procedure allowed us to recover different CTCs phenotypes and to link their gene expression profile with the clinical features of the patients and CTCs account. 

## 2. Results

### 2.1. EpCAM+ CTCs Enumeration Monitoring

**Figure 1:**
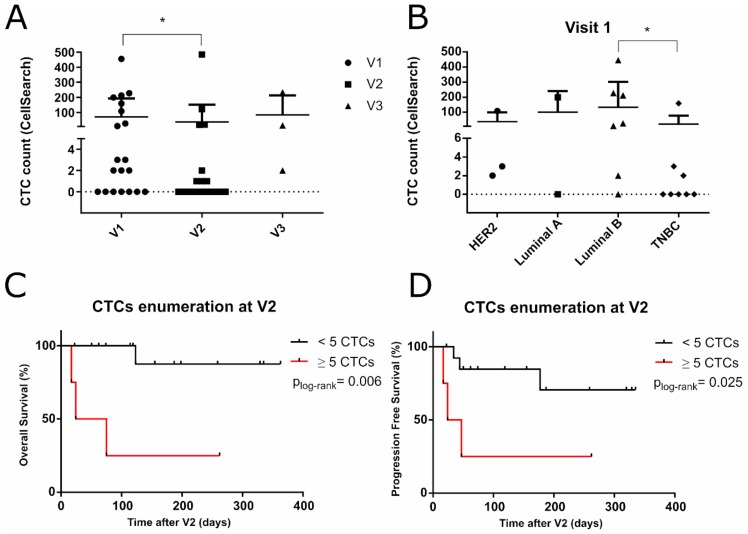
(**A**) Longitudinal CTCs enumeration by CellSearch^®^ on the BC patient cohort, (V1 mean = 69.85, range = 0–445, V2 mean = 35.9, range = 0–484, V3 mean = 83, range = 2–233; Wilcoxon test, *p* = 0.041). (**B**) Representation of CTCs enumeration across the different molecular subtypes of BC at V1 (Mann Whitney test, *p* = 0.032) with mean values: luminal A (99.5, range = 0–199), luminal B (131.6, range = 0–445), HER2 over-expressed (37.7, range = 2-108), TNBC (20.5, range = 0–159) (**C**–**D**) Estimates of probabilities for OS and PFS at V2 (49.5 days, *p* = 0.006 and 35.5 days, *p* = 0.025) in advanced and metastatic BC patients with ≥5 CTCs or <5 CTCs per 7.5 mL of blood. CTC: circulating tumor cells, BC: breast cancer, HER2: human epidermal growth factor receptor 2, TNBC: triple negative BC, OS: overall survival, PFS: progression-free survival (* <0.05; ** <0.001; *** <0.0001).

For CTCs enumeration, 41 blood samples were collected and analyzed by CellSearch (at the diagnosis of metastasis before chemotherapy, visit 1 or V1 = 20; after one month of treatment, visit 2 or V2 = 18 and after the clinical progression of the disease, visit 3 or V3 = 3). At the diagnosis of metastasis (V1), 65% of the patients showed detection of CTCs (≥1 CTCs), and 40% were classified as bad prognosis based on the predefined cut off ≥5 CTCs/7 mL of blood (mean = 174.4, range = 9–445). After the first cycle of treatment, at V2, the percentage of patients with ≥5 CTCs was 22% (mean = 160.5, range = 18–484), and 10 out of the 11 CTC positive samples at V1 suffered a reduction on their CTCs counting (Wilcoxon test, *p* = 0.041). At V3, two out of the three patients that progressed showed ≥5 CTCs, and one of the samples displayed a high increase in the CTCs account from 121 (V2) to 233 CTCs (V3) (mean = 123.5, range = 2–233) (Figure 1A).

To study if CTCs detection was more frequent in a specific subtype, we performed the analysis of CTCs enumeration data by molecular subtypes. This analysis revealed that at baseline (V1), CTCs detection by CellSearch^®^ occurred mainly in luminal and HER2 patients, while CTCs detection in TNBC patients was rare. Indeed, CTC detection between luminal B and TNBC at V1 was statistically different (Figure 1B). In addition, patients with ≥5 CTCs regarding the different subtypes were 66.6% of luminal patients, 12.5% TNBC patients, and 33% HER2 patients. At V2, luminal cases maintained more CTCs than the other subtypes; however, no significant differences were detected.

Next, in order to examine the prognostic value of CTCs enumeration in our cohort, we performed a survival analysis considering the cut off ≥5 CTCs. Patients with ≥5 CTCs at V1 showed a poorer outcome, although significant differences were not found. Interestingly, at V2, after the first cycle of therapy, patients with ≥5 CTCs had both shorter PFS and OS (Figure 1C,D). There were not enough samples at the clinical progression time point (V3) to perform conclusive survival analysis. TNBC patients showed a worse outcome when they had ≥5 CTCs for both visits (Figure S1).

### 2.2. Unbiased CTC Gene Expression For Advanced BC Patients Monitoring

**Figure 2:**
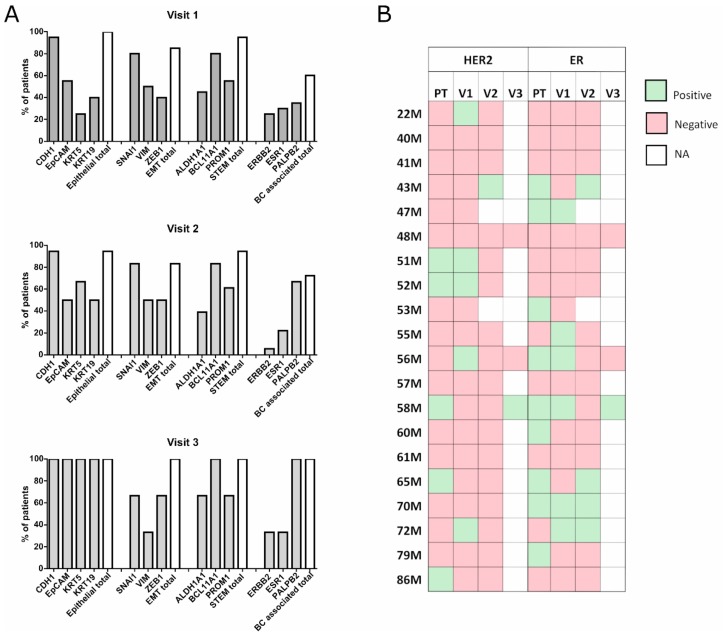
(**A**) Percentages of patients showing positive expression (fold change ≥1.5) for the epithelial, EMT, stem, and BC-associated genes in the different time points of the disease. A white bar is showing the percentage of patients expressing the total number of markers for each gene group analyzed. (**B**) HER2 and ER expression evolution on the primary tumor and the CTCs at the different time points of the disease. PT: primary tumor, V1: Visit 1, V2: Visit 2, V3: Visit 3 (positive expression in green, negative in red, deceased or not available (NA) patients in white). EMT: epithelial-mesenchymal transition, ER: estrogen receptor.

Next, the gene expression of CTCs from each patient was calculated relative to the autologous peripheral blood mononuclear cells (PBMCs) expression, minimizing the bias from unspecific isolation of blood cells. For this analysis, we considered fold change ≥1.5 as positive expression, and we found that in all visits, at least one epithelial marker was detected in all patients, being *CDH1* the most commonly expressed gene in the analyzed patients (95%, 95%, and 100% in V1, V2, and V3, respectively). Regarding the EMT markers, their expression was highly homogeneous between all the visits, with *SNAI1* the most frequently expressed (80%, 83%, and 64%, respectively, for V1, V2, and V3). At least one BC-associated maker (*ERBB2*, *ESR1*, *PALB2*) was detected in 60% of the patients at V1, 72% at V2, and 100% of the patients at V3 (Figure 2A).

For HER2 and ER expression (positive or negative) in the primary tumor, we checked the pathology report to liken with the expression of *ERBB2* and *ESR1* in the CTCs at the three different visits. We found concordance in the HER2 status in 70% of the patients at V1, 55.5% at V2, and 66% at V3 (Figure 2B). Interestingly, four patients with HER2-tumors had *ERBB2+* CTCs. Regarding ER expression, we detected concordance with the primary tumor in 65% of the patients at V1, 66.6% at V2, and 100% at V3. Just like *ERBB2*, *ESR1* expression showed a changing profile of expression in the different time points of the disease.

### 2.3. CTC Detection By Cell Search and Gene Expression Profiles

To define high/low levels of each marker, the median expression values of each gene for every visit were considered (above or below) and were associated with CellSearch^®^ (Menarini, Silicon Biosystems Inc, Bologna, Italy) enumeration.

The expression profile of CTCs at V1 that associates significantly with CTCs detection by CellSearch^®^ was defined by *ERBB2* and *EpCAM* positive expression, regardless of CTCs accounts (≥1 CTCs or ≥5 CTCs). Those cases without CTCs detected by CellSearch^®^ (CTCs = 0) showed a more stem/EMT profile (considering the high expression of at least one gene of the same functional group, see Table S1). In addition, high expression levels of *GDF15* and *ESR1* were significantly associated with CTCs detection by CellSearch (≥1 CTCs). When considering the cut off ≥5 CTCs, this association was also established with high expression levels of *KRT19* (Table 1). Besides, 89% of the patients whose CTCs enriched fraction lacked *EpCAM* expression were CTCs negative by CellSearch. However, we found the expression of other epithelial markers in these samples (*CDH1* and *KRT19*). Furthermore, after one cycle of treatment, at V2, the high expression of the cell cycle-related gene *CCND1* (which encodes Cyclin D1) was associated with the detection of ≥5 CTCs (*p* = 0.02).

### 2.4. CTCs Expression Profile and Clinical Data

Compared with TNBC subtype, luminal tumors showed higher expression levels of *KRT19* in CTCs isolated from V1 (Kruskal–Wallis test, *p* = 0.005) and lower *CDH1* in V2 (*p* = 0.012). Interestingly, the presence of estrogen receptor (ER+) was associated with low expression of *RB1* (*p* = 0.025) and high expression of *KRT19* (*p* = 0.002) in V1, which was consistent with a higher expression of *KRT19* in luminal subtypes. On the contrary, PR-patients showed a high expression of *RB1* together with *BCL11A* and *KRT5* (*p* = 0.019) in CTCs from V1.

Patients diagnosed with more advanced tumor grades at metastases showed CTCs with high expression levels of *KRT5* and low expression of *E2F4* (*p* = 0.024). CTCs from patients who had undergone surgery were associated with high expression of *KRT19*, *EpCAM*, and *ESR1* genes (*p* = 0.025).

Next, we studied if CTCs expression at V1 was associated with distal metastasis localization at the diagnosis of metastasis. CTCs with low expression of *VIM* (*p* = 0.015) and *CCND1* (*p* = 0.004) were detected in patients with lung metastasis. Low expression of *VIM* and *RB1* (*p* = 0.020) but high *ESR1* expression (*p* = 0.020) was identified in CTCs’ patients who had bone metastasis. Lastly, high levels of *PALB2* expression were associated with liver metastasis (*p* = 0.04) and lymph nodes affectation (*p* = 0.005) (Figure S2). In addition, *CDK4* expression in the primary tumor (formalin-fixed, paraffin-embedded tissues (FFPA) samples, *n* = 8) was higher in those patients that did not respond to therapy (*p* = 0.028).

**Figure 3:**
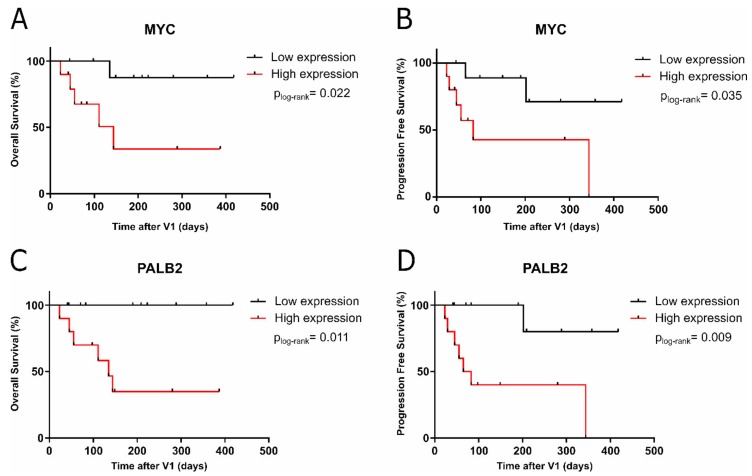
Kaplan–Meier plots for (**A**) OS based on the expression of *MYC* (144 days, *p* = 0.022); (**B**) PFS based on the expression of *MYC* (83 days, *p* = 0.035); (**C**) OS based on the expression of *PALB2* (135 days, *p* = 0.011); and (**D**) PFS based on the expression of *PALB2* (74 days, *p* = 0.009) at V1.

To identify markers with prognostic value, we performed survival analysis. On the CTCs enriched fraction, results showed that, at V1, high expression levels of *MYC* and *PALB2* were able to discriminate patients with poor outcomes (Figure 3). Thereby, patients whose CTCs showed higher expression levels of *MYC* and *PALB2* were linked to shorter OS and PFS.

Lastly, we performed a comparison among visits, finding that after 1 cycle of treatment, *PALB2* expression on CTCs was increased (*p* = 0.03).

### 2.5. EpCAM^high^VIM^low^ALDH1A1^high^ CTCs Signature Predicts Shorter Outcome

**Figure 4:**
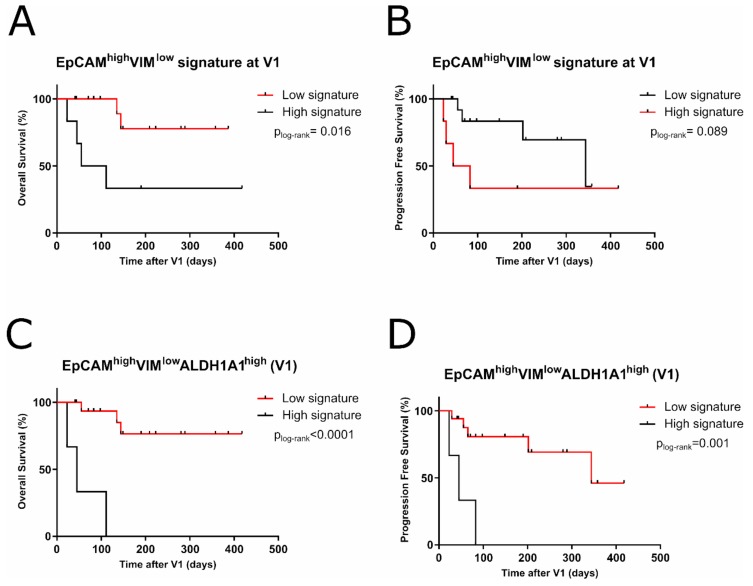
(**A**,**B**) Kaplan–Meier plot for OS and PFS based on the expression of *EpCAM*^high^*VIM*^low^ signature (83 days, *p* = 0.016 and 64 days, *p* = 0.089, respectively). (**C**,**D**) Kaplan–Meier plot for OS and PFS of the *EpCAM*^high^*VIM*^low^*ALDH1A1*^high^ signature (45 days, *p* < 0.0001 and 45 days, *p* = 0.001, respectively).

The isolation of CTCs was performed using a negative enrichment protocol in order to recover a wider CTCs population. High expression levels of *EpCAM* at V1 showed a tendency to discriminate patients with worse prognosis but without statistical significance. In addition, we found that high expression of *VIM*, EMT-related gene, led to a better outcome in the patients analyzed, thus larger OS (*p* = 0.011). Next, we established a signature considering both expression data, that is, high expression of *EpCAM* combined with low expression of *VIM*. For this analysis, all the samples collected at V1 were included, regardless of their CellSearch counting or their *EpCAM* expression level. This *EpCAM*^high^*VIM*^low^ signature was able to predict a shorter OS (83 days, *p* = 0.016) at V1 (Figure 4A). However, it did not improve the significance of *VIM*^low^ separately (135 days).

If we considered the CTCs with stem phenotype by including high *ALDH1A1* expression in the signature, *EpCAM*^high^*VIM*^low^*ALDH1A1*^high^, we were able to discriminate those patients with poorer outcome, improving the statistical power of the analysis (OS, 45 days, *p* < 0.0001 and PFS, 45 days, *p* = 0.001) (Figure 4C,D). *ALDH1A1* expression alone was not able to discriminate patients with poor prognosis (OS and PFS *p* > 0.05).

## 3. Discussion

Through liquid biopsy, CTCs can be sampled and characterized over time during the metastatic disease in order to monitor treatment response and disease progression. In addition, cancer cells in circulation can complete the understanding of the metastatic cascade [12]. In this study, we used a negative enrichment protocol to isolate CTCs from advanced and metastatic BC patients, allowing the enrichment of CTCs from all phenotypes, including epithelial CTCs and also CTCs in a more mesenchymal or stem state [13]. Furthermore, we enumerated the CTCs of each sample by CellSearch^®^.

Our results showed that patients with ≥5 CTCs showed a lower OS, although not reaching the statistical significance considering the metastatic diagnosis time point (V1). These data differed with previously described reports where patients with ≥5 CTCs were related to the worst prognosis [8]. We found that 40% of the analyzed patients showed ≥5 CTCs at V1 (counted by CellSearch^®^). This was a slightly lower percentage than that previously described by Lianidou and colleagues (50–70%) [14]. These discrepancies could be due to the differences in subtype proportions within cohorts and the reduced number of cases. Specifically, our cohort included a high proportion of TNBC cases, which usually present lower EpCAM+ CTCs counts due to their mesenchymal or stem features [15]. Regarding the overall distribution of CTCs in different subtypes, a larger proportion of patients in the luminal subset was CTCs+ by CellSearch^®^, compared with the other tumors subtypes, as previously described by Giordano and colleagues [16]. Although the TNBC subtype lowered CTCs counts, we found that the ≥5 CTCs cut off in TNBC patients was enough to predict poor OS.

Interestingly, after one cycle of treatment (V2), the enumeration of ≥5 CTCs in patients was also significantly associated with worse OS and PFS. Although, several studies have evaluated the prognostic value of CTCs enumeration in BC patients [8,17,18], only a number of them have explored the prognostic relevance of CTCs numbers before and after chemotherapy, supporting that the presence of persisting CTCs after treatment is associated with worse outcome, and that a decrease in the CTCs count is an early marker of individual response, during the follow-up [19,20].

Besides the prognostic impact of CTCs enumeration, the molecular characterization of these cells offers new perspectives that can increase the understanding of CTCs biology and can enable better clinical decisions, which nowadays are not possible through enumeration. In this sense, some studies have explored CTCs in BC by using molecular methods [21,22,23,24,25,26,27,28]. However, only a few have evaluated the characteristics of CTCs after therapy [19,26,29,30,31], and, to our knowledge, only one has used a negative enrichment approach comparable with ours [32].

Compared with previous articles [23,25,32,33], we reported a higher detection rate of epithelial, EMT, and stem markers expression on CTCs, probably due to the isolation method of choice. In addition, compared to the study of Aaltonen and colleagues, we observed discordances in the ER expression between the patient’s solid tumor and CTCs [34], emphasizing the dynamic nature of tumors and the need for real-time monitoring in cancer patients.

Our molecular analyses revealed that the expression of specific epithelial (*EpCAM*, *KRT19*) or BC-related genes (*ERBB2*) was associated with the presence of ≥5 CTCs at V1. These data showed that the isolation of CTCs occurred in a specific manner and that the epithelial population isolated with the CellSearch^®^ approach was also harvested with this strategy. Furthermore, Bredemeier and colleagues described *KRT19* as a powerful marker to identify CTCs in metastatic breast cancer (MBC) [31]. We also found that *KRT19* was higher in luminal patients versus TNBC. These results could be explained by the fact that *KRT19* encodes for CK19 protein, an epithelial marker which can be downregulated during the EMT process [35]; hence, it is lowly expressed in the TNBC subtype [36].

*CCND1* oncogenic capacity has long been established in BC, and its overexpression in transgenic mammary tissues has been linked with mammary hyperplasia and tumors [37]. Our results suggested that those patients that did not respond to therapy (≥5 CTCs in V2) showed a higher *CCND1* gene expression. Since Cyclin D1 plays a central role in the regulation of proliferation, these CTCs could be linked with treatment failure and might be relevant for CDK4/6 inhibitor therapies.

Analyzing clinical data in a comprehensible manner, we found an association between higher tumor grade at diagnosis and overexpression of *KRT5* in CTCs at V1, which is the gene that encodes CK5. CK5-positive BC cells have enhanced mammosphere forming potential and are endocrine and chemotherapy-resistant in MBC [38]. However, *KTR5* expression has not been reported yet on CTCs. Although there is no conclusive data regarding keratin expression in CTCs, BC cells show a complex pattern of keratin expression, and nowadays there is not a good candidate to identify CTCs. Our data supported that, for immunophenotyping, the use of antibody cocktails instead of individual keratin antibodies is recommended [39]. Interestingly, those patients who underwent surgery showed CTCs with more epithelial phenotype (*KRT19* and *EpCAM*), which might be explained by the presence of cells that were shed from primary tumor in the intervention, in accordance with Alieva and colleagues, who proposed a potential impact of invasive surgical procedures on primary tumor growth and metastasis [40].

The expression of the progesterone receptor (PR) is highly estrogen-dependent and has, therefore, been linked to endocrine therapy sensitivity prediction [41]. We found high expression levels of *RB1*, *BCL11A,* and *KRT5* in patients diagnosed with PR-negative tumors, which might suggest their participation in endocrine resistance in some luminal cases. BCL11A has also been identified in aggressive subtypes of BC, and its overexpression drives the development and progression of TNBC, as seen by Khaled and colleagues [42] and Abreu and colleagues (unpublished data). Regarding CK5, Dairkee and colleagues reported for the first time the possible poor survival or early recurrence associated with the expression of CK5 in tumor cells in 1987 [43] and, although the functional role of the CK, such as CK5, CK14, or CK17, is still unknown, their expression is clearly associated with poor prognosis.

The low *RB1* expression observed in CTCs from ER+ patients is in agreement with previous reports, where RB functional status seems to be linked to response to endocrine therapy [44]. This supports that RB is only infrequently lost in ER-positive breast cancer, and its loss decreases the expression of *ESR1* gene [45]. All these data have been reported in tissue, and no data regarding CTCs have been published so far. In summary, we found that primary tumors with different hormonal receptor characteristics led to CTCs with different expression profiles.

Our data remarked the potential of CTCs analysis for patients’ prognosis since different gene expression profiles correlate with the patient’s outcome. *MYC* encodes the oncoprotein c-MYC, and its overexpression is associated with poor clinical outcomes in BC patients [46]. Little is known regarding its expression profile in MBC CTCs; nonetheless, *MYC* inhibitors have been proposed for targeting cancer stem cells in drug-resistant TNBC [47]. In addition, *MYC* has been related to anti-estrogen therapy resistance [48]. Here, we reported that the tracking of *MYC* expression in CTCs was feasible and it might be of interest in BC patients, in agreement with Reinhardt and colleagues, who described high *MYC* expression in CTCs’ differential phenotypes in BC [49]. Regarding *PALB2* (Partner And Localizer of Breast Cancer Type 2 susceptibility protein (*BRCA2*)), mutations in this gene have been associated with increased risk of BC [50]. *PALB2* acts as a tumor suppressor protein by mediating DNA repair and thereby suppressing genome instability [51]. In addition, its overexpression in tissue has recently been linked to a worse outcome in patients with MBC [52]. We found similar results on CTCs concerning patients’ outcomes and found higher expression after one cycle of therapy, which might be induced to promote DNA repair. These data allow real-time monitoring of *PALB2* status in the patients and efficacy prediction to Poly (ADP-ribose) Polymerases (PARPs) inhibitors, not described so far [53].

In our analysis, we found that low expression of *VIM*, a mesenchymal marker, was correlated with a poorer outcome on the patients. In addition, an *EpCAM*^high^*VIM*^low^ signature could predict the worst outcome with better significance than EpCAM expression alone. Polioudaki and colleagues described that CTCs undergoing EMT acquired mesenchymal morphology, which was associated with full or partial CK19 replacement by Vimentin [35]. The EMT status of CTCs has been a matter of controversy, with some studies pointing to an association between tumor cells with partial EMT state and a worse outcome, when compared with cells that have undergone complete EMT [54,55,56,57]. In addition, it has been postulated that EMT is not enough for metastasis in a number of cancer types [58,59,60], and a recent publication by Padmanaban and colleagues described that E-Cadherin was required for metastasis [61], reinforcing the role of epithelial markers in this process. Some of the studies made on CTCs may be biased by the isolation method used, which is mostly based on a selection marker or a combination of several markers. Interestingly, Markiewicz and colleagues, with a negative enrichment approach based only on anti-CD45 magnetic beads, found that an EMT subtype of CTCs did not have any significant impact on the survival of early BC patients [32]. Furthermore, a recent study by de Wit and colleagues on breast and prostate cancer patients reported that the presence of *EpCAM*^low^ CTCs had no relation with OS [62]. Additionally, when we included *ALDH1A1* expression in the signature, *EpCAM*^high^*VIM*^low^*ALDH1A1*^high^, its outcome prediction potential power was improved, remarking the need for the study of more cells markers besides *EpCAM*. In addition to its association with therapy failure, *ALDH1A1* expression in tissue samples has been related to poor prognosis in different BC subtypes [63,64,65,66], and its expression in CTCs has also been linked with worse outcome in BC patients [23,26,28,32,67]. Likewise, Keup and colleagues suggested that the use of novel agents to attack BC stem cells, like salinomycin and a new synthetic curcumin analog against Aldehyde Dehydrogenase 1 (ALDH1), could be promising in patients with ALDH1+ CTCs [27]. In addition, a recent publication describes that EpCAM+ cells with stem phenotypes provide prognostic information in early-stage breast cancer patients [68], reinforcing our findings.

We demonstrated that CTCs molecular characterization improved the clinical value of CTCs enumeration; however, the transcriptome analysis of CTCs at a single cell level could allow the precise determination of the epithelial/mesenchymal/stem state of each cell, providing better insight into CTCs heterogeneity and its significance in patients’ prognosis.

## 4. Materials and Methods

### 4.1. Patients

A cohort of 20 patients (median age 53 years) diagnosed of advance or metastatic BC at the Clinical Hospital of Santiago de Compostela (Spain) was included in the study from August 2016 to July 2018 (Table S2). Patients were enrolled in the study after informed consent and following the approval and recommendations of the Ethics Committee of Galicia (code: 2015/772). This cohort was distributed as follows: 10% of the patients were luminal A, 35% were luminal B subtype, 40% were TNBC, and 15% were HER2-overexpressed subtype.

Inclusion criteria comprised of patients with advanced breast cancer, of any subtype, with indication to initiate first-line of palliative, endocrine, cytotoxic, and/or biological systemic treatment; disease-free interval ≥12 months; age ≥18 years; informed consent dated and signed; followable disease (measurable or not); ability and willingness to follow the study plan; PS-ECOG 0-3; and adequate function of organs to receive antineoplastic treatment. Exclusion criteria were known diagnosis of psychiatric illness that contraindicates antineoplastic treatment or prevents the patient from understanding and accepting the conditions of the study; radiation therapy on follow-up injury (if < than 6 months); current or malignant tumor in the last 5 years, except for basal cell carcinoma or squamous cell carcinoma or carcinoma in situ of the cervix properly treated.

### 4.2. Clinical Samples

Two 10 mL tubes of peripheral blood in EDTA-coated vacutainers (Becton Dickinson, Franklin Lanes, NJ, USA) were obtained for each patient at diagnosis of metastasis before chemotherapy (Visit 1, V1; *n* = 20), after one cycle of therapy (Visit 2, V2; *n* = 18; 2 patients deceased before V2 sample collection) and after clinical progression (Visit 3, V3; *n* = 3). Blood samples were processed within two hours after withdrawal. For CTCs enumeration purposes, one additional 10 mL CellSave Preservative tube (Menarini-Silicon Biosystems, Bologna, Italy) was obtained in parallel at each withdrawal, and it was analyzed at the Liquid Biopsy Analysis Unit of the Clinical Hospital of Santiago de Compostela (Santiago de Compostela, Spain). In 8 patients, formalin-fixed paraffin-embedded (FFPE) samples from normal tissue and the primary tumor were provided by the Pathology Service and the BioBank of the Clinical Hospital of Santiago de Compostela (PT17/0015/0002), integrated into the Spanish National Biobanks Network. All samples were anonymized and encoded before the analysis.

### 4.3. CTC Enumeration

Blood samples collected in CellSave tubes were analyzed for CTC enumeration by the CellSearch^®^ System, using CellSearch Epithelial Circulating Tumor Cell Kit (Menarini, Silicon Biosystems Inc, Bologna, Italy). EpCAM-positive enriched cells were labeled with phycoerythrin-conjugated anti-cytokeratins (8, 18, and 19) antibodies, allophycocyanin-conjugated anti-CD45 antibodies, and with 4,6-diamino-2-phenylindole (DAPI). The CellTracks Analyzer (Menarini-Silicon Biosystems, Bologna, Italy) was used to acquire digital images of the 3 different fluorescent dyes, which were reviewed by trained operators to determine the CTCs count.

### 4.4. Peripheral Blood Mononuclear Cells (PBMCs) Isolation and CTCs Enrichment

PBMCs isolation was performed from 10 mL of peripheral blood (collected using an EDTA tube) by density gradient centrifugation protocol (LymphoprepTM, STEMCELL Technologies, Vancouver, BC, Canada) in SepMate™ tubes (STEMCELL Technologies, Vancouver, BC, Canada) according to manufacturer’s instructions. Another 10 mL of peripheral blood was used to collect CTCs by negative selection using the RosetteSep™ CTC Enrichment Cocktail Containing Anti-CD56 (STEMCELL Technologies, Vancouver, BC, Canada) according to the manufacturer’s protocol. Enriched cells were placed in RNAlaterTM Solution (Invitrogen, ThermoFisher Scientific, Carlsbad, CA, USA) and kept at −80 °C until further analyses.

### 4.5. Nucleic Acid Extraction, cDNA Synthesis, Preamplification, and Quantitative Real-Time PCR (qPCR)

The extraction of RNA from the enriched tumor cells and the PBMCs was performed using AllPrep DNA/RNA Mini Kit (Qiagen, Valencia, CA, USA) following the manufacturer’s protocol. RNA from FFPE tissue sections was extracted with the miRNeasy FFPE kit (Qiagen, Valencia, CA, USA). According to the manufacturer’s instructions, 11 µL of total RNA was retrotranscribed into cDNA using the SuperScript III (Thermo Fisher Scientific, Carlsbad, CA, USA). Due to the low recovery of RNA, samples were preamplified with Taqman Preamp Master Mix (Thermo Fisher Scientific, Carlsbad, CA, USA). cDNA expression was analyzed on a LightCycler 480 II (Roche Diagnostics, Indianapolis, IN, USA) with TaqMan Gene Expression Master Mix and TaqMan probes (Applied Biosystems, Carlsbad, CA, USA) for a customized panel of 25 genes (Table S2). *B2M* was used as a reference gene, and, after data normalization, gene expression from CTCs was relativized to the autologous PBMCs transcripts. Primary tumor expression was relativized to the corresponding normal tissue.

### 4.6. Statistical Analysis

Statistical analysis was performed using GraphPad Prism 6.01 software (GraphPad Software Inc., La Jolla, CA, USA), IBM SPSS Statistical Software (version 10.0, IBM Corp, Chicago, IL, USA), and R Studio (Version R-3.5.0, https://www.R-project.org/). The Wilcoxon signed-rank test was used to compare CTCs longitudinal enumeration and Mann–Whitney test for comparison among visits. Assuming PBMCs contamination in the enriched fraction of CTCs, the expression of the autologous PBMCs was used as a normalizer. Differences in CTCs enumeration and expression among subtypes were compared with the Kruskal–Wallis test. Fisher test and Mann–Whitney test were used to study the association between enumeration and gene expression of the CTCs. Correlations between gene expression and clinical data were tested by Fisher’s exact test. Progression-free survival (PFS) and overall survival (OS) were visualized using Kaplan–Meier plots and tested by the log-rank test. Only *p*-values < 0.05 were considered statistically significant.

## 5. Conclusions

Our data suggested that an epithelial-stem state of the CTCs might give rise to a more aggressive disease. In any case, when analyzing CTCs as bulk, the chosen detection method for the isolation of CTCs is decisive, as some methods may underestimate or neglect certain subpopulations of CTCs with putative relevant roles.

All in all, our promising results corroborated that CTCs phenotypes could provide clinically important information regarding patients’ survival and their stratification into specific clinical studies. The CTCs population is a heterogeneous one with different subpopulations that could lead to different prognosis throughout the disease.

## Figures and Tables

**Table 1:** Patient’s classification by CTCs (circulating tumor cells) account and gene expression.

**CTCs**	**Gene Expression (*ERRB2*, *EpCAM*, *ESR1*, *GDF15*)**		***p*-Value**
	**High**	**Low**	
**0 CTCs**	1	6	0.019
>**1 CTCs**	9	4	
	**Gene Expression (*ERRB2*, *EpCAM*, *KRT19*)**		***p*-Value**
	**High**	**Low**	
<**5 CTCs**	3	9	0.006
≥**5 CTCs**	7	1	

## Supplementary Materials

The following are available online at https://www.mdpi.com/2072-6694/11/12/1941/s1, Figure S1: Estimates of probabilities for OS and PFS at V1 (OS: 55 days, *p* = 0.008; PFS: 29 days, *p* = 0.008) and V2 (OS: 17 days, *p* = 0.008; PFS: 17 days, *p* = 0.008) in TNBC patients with ≥5 CTCs or <5 CTCs per 7.5 mL of blood. Figure S2: Representation of high or low expressed genes on the CTCs isolated fraction depending on the metastasis site. Table S1: Gene Expression Custom Panel Assay for The Study of CTCs On the BC Patient’s Cohort. Table S2: Clinical Parameters of The BC Patients Included in The Study.
