# Supplementary material for: CTCs Expression Profiling for Advanced Breast Cancer Monitoring
Source: Cancers (Basel). 2019 Dec 4;11(12):1941. doi: 10.3390/cancers11121941 (PMC6966538; doi:10.3390/cancers11121941)
Supplement: Supplementary file 1 [file cancers-11-01941-s001.pdf]

Supplementary Material

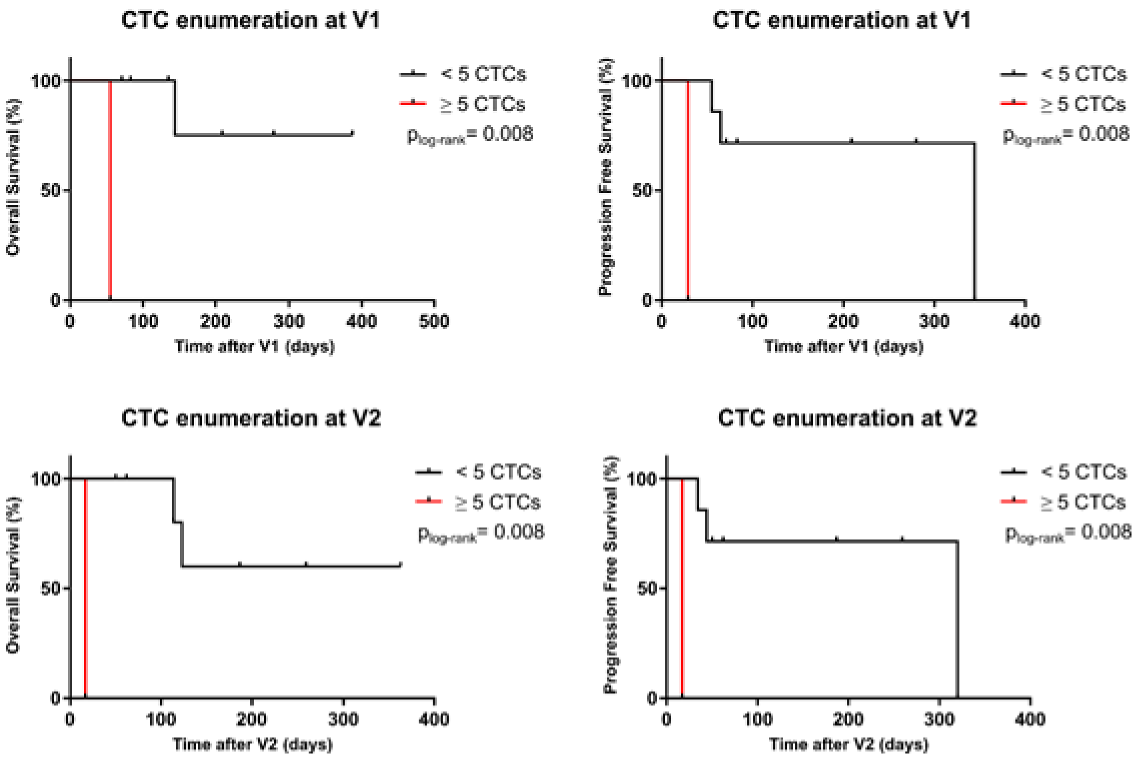

Figure S1. Estimates of probabilities for OS and PFS at V1 (OS: 55 days, $p = 0.008$; PFS: 29 days, $p = 0.008$) and V2 (OS: 17 days, $p = 0.008$; PFS: 17 days, $p = 0.008$) in TNBC patients with ≥ 5 CTCs or < 5 CTCs per 7.5 ml of blood.

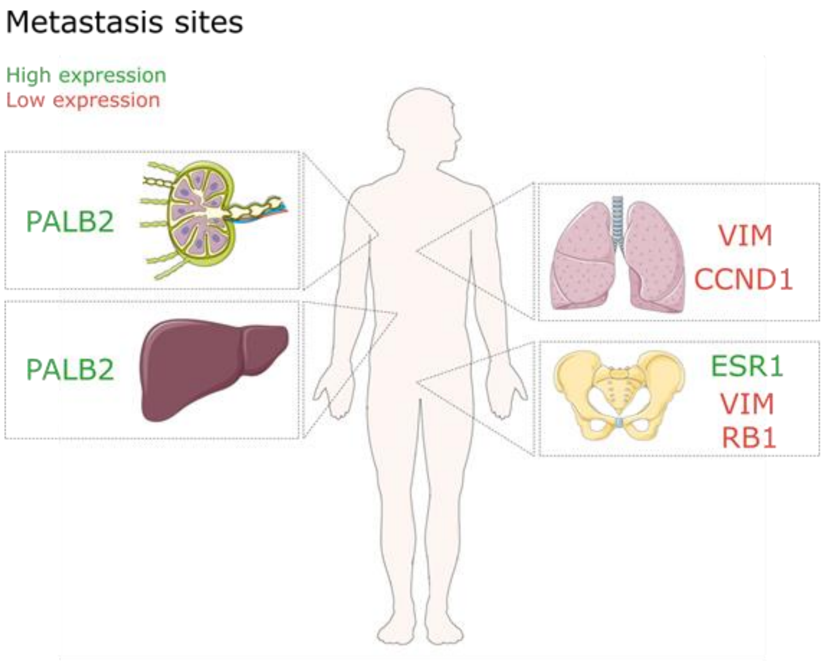

Figure S2. Representation of high or low expressed genes on the CTCs isolated fraction depending on the metastasis site.

Table S1. Gene Expression Custom Panel Assay For The Study of CTCs On The BC Patient´s Cohort.

Gene name	Taqman Assay	Functional Gene Grouping
ALDH1A1	Hs00946916_m1	Stem
B2M	Hs00187842_m1	Housekeeping gene
BCL11A1	Hs01093197_m1	Stem
BCL2	Hs00608023_m1	Apoptosis
CCND1	Hs00765553_m1	Proliferation/cell cycle regulation
CD36	Hs00354519_m1	Cell metabolism
CDH1	Hs00170423_m1	Epithelial
CDK4	Hs01565683_g1	Proliferation/cell cycle regulation
CTNNB1	Hs00355049_m1	Cell adhesion/gene transcription
E2F4	Hs00608098_m1	Proliferation/cell cycle regulation
EpCAM	Hs00158980_m1	Epithelial
ERBB2	Hs01001580_m1	Breast cancer associated
ESR1	Hs01046816_m1	Breast cancer associated
FAS	Hs00163653_m1	Apoptosis
GDF15	Hs00171132_m1	Proliferation/cell cycle regulation
KRT5	Hs00361185_m1	Epithelial
KRT19	Hs00761767_s1	Epithelial
MYC	Hs00153408_m1	Oncogene
MYCL	Hs00420495_m1	Oncogene
PALPB2	Hs00226617_m1	Breast cancer associated
PROM1	Hs01009257_m1	Stem
PTPRC	Hs04189704_m1	Blood cell
RB1	Hs01078066_m1	Proliferation/cell cycle regulation
SNAI1	Hs00195591_m1	EMT
VIM	Hs00958116_m1	EMT
ZEB1	Hs00232783_m1	EMT

Table S2. Clinical Parameters of The BC Patients Included In The Study.

**Table I. Clinical parameters**	
**Variable**	**n(%)**
Age	
> 50 years	14 (70%)
≤ 50 years	6 (30%)
Tumour Stage	
IV	20 (100%)
ER status	
Positive	8 (40%)
Negative	12 (60%)
PR status	
Positive	7 (35%)
Negative	13 (65%)
HER2 status	
Positive	6 (30%)
Negative	14 (70%)
Metastasis location	
Bone & Visceral	10 (50%)
Visceral	9 (45%)
Unknown	1 (5%)
**Total**	**20 (100%)**
